# Triglyceride to high-density lipoprotein cholesterol ratio was negatively associated with relative grip strength in older adults: a cross-sectional study of the NHANES database

**DOI:** 10.3389/fpubh.2023.1222636

**Published:** 2023-11-02

**Authors:** Yan Huang, Jian Liao, Yang Liu

**Affiliations:** Department of Medical Laboratory, The First Affiliated Hospital of Traditional Chinese Medicine of Chengdu Medical College, Chengdu, China

**Keywords:** TG/HDL-C ratio, relative grip strength, NHANES, older adults, sarcopenia

## Abstract

**Aim:**

This study aims to explore the association between triglyceride to high-density lipoprotein cholesterol (TG/HDL-C) ratio and relative grip strength in older adults in order to provide some references for the prevention and control of sarcopenia.

**Methods:**

For this cross-sectional study, the demographic and clinical data of 1,404 individuals aged ≥60 years old were extracted from the National Health and Nutrition Examination Survey (NHANES) database in 2011–2014. The definition of relative grip strength was the sum of the largest reading from each hand/body mass index (BMI) ratio. We used weighted univariate linear regression and stepwise regression analysis to screen the covariates. Weighted univariate and multivariate linear regression analyses were used to explore the association between the TG/HDL-C ratio and the relative grip strength. We also explored this relationship in subgroups of gender, diabetes mellitus (DM), cardiovascular disease (CVD), and arthritis. The evaluation index was β with 95% confidence intervals (CIs).

**Results:**

A total of 1,306 older adults were eligible. After adjusting for the covariates including age, gender, race, marital status, physical activity, DM, CVD, arthritis, and chronic kidney disease (CKD), we found that the TG/HDL-C ratio was negatively linked to the relative grip strength (all *p* < 0.05). Furthermore, the increased TG/HDL-C ratio was also related to the decreased relative grip strength in those who were women, not having DM, and having CVD (all *p* < 0.05).

**Conclusion:**

With the increase in the TG/HDL-C ratio, the relative grip strength of older adults decreased significantly, indicating that the TG/HDL-C ratio could be closely monitored in the older adult population and may be associated with the prevention and control of sarcopenia.

## Introduction

Sarcopenia is a progressive and widespread skeletal muscle disease and the risk increases with age (1). It is estimated that sarcopenia has impacted 10–16% of older adults worldwide (2). Sarcopenia increases the risk of adverse consequences such as falls, fractures, physical disabilities, and death in older adults which causes a substantial increase in economic burden on patients’ families and society (3).

Low muscle strength is the primary parameter of sarcopenia because it is the most reliable indicator of muscle function (4). Grip strength is one of the most common muscle strength markers in clinical and research settings (5). Grip strength and sarcopenia in older adults have important implications for predicting multiple diseases and the risk of death. A large-sample prospective study of UK Biobank participants found that lower grip strength was associated with a higher risk of developing severe non-alcoholic fatty liver disease (3). Another study in community-dwelling older adults showed that screening for sarcopenia severity and low muscle strength may be used to identify the risk of cognitive impairment (6). In addition, Takahashi et al. (7) indicated that sarcopenia was associated with incident all-cause mortality in older outpatients with type 2 diabetes mellitus (T2DM).

Muscle activity depends on glucose metabolism, and insulin resistance (IR) can decrease the muscle’s ability to utilize glucose, reduce intracellular energy production, and weaken muscle strength (8). The homeostasis model assessment of insulin resistance (HOMA-IR) is a traditional IR index with limited clinical application (9). However, the triglyceride to high-density lipoprotein cholesterol (TG/HDL-C) ratio has been suggested as a new alternative IR index in recent years (10), which has been also applied to research studies in diabetes mellitus (DM) (11) and cardiovascular disease (CVD) (12). A recent study in older Korean men showed that the TG/HDL ratio was positively related to a higher risk of sarcopenia (13). Another study in community-dwelling Chinese adults also found a negative association between the sarcopenia occurrence rate and the TG/HDL-C ratio (14).

Measures of grip strength are convenient assessments of strength capacity and a reliable measure of muscle function (15, 16). However, studies on the relationship between the TG/HDL-C ratio and muscle function are still absent, and the results on the role of the TG/HDL-C ratio in sarcopenia were inconsistent. Herein, this study aims to explore the association between TG/HDL-C ratio and relative grip strength in individuals aged ≥60 years old and further discusses this relationship in those who are of different genders as well as the complications. We hope that this study can provide some references for the prevention and management of sarcopenia in older adult populations.

## Methods

### Study design and population

Data in this cross-sectional study were extracted from the National Health and Nutrition Examination Survey (NHANES) database from 2011 to 2014. NHANES is a multipurpose research program conducted by the National Center for Health Statistics (NCHS) to assess the health and nutritional status of adults and children in the United States. More details are present on the NHANES website: https://wwwn.cdc.gov/Nchs/Nhanes/. The survey regularly collects data from approximately 5,000 persons from 15 areas since 1999, which includes a household interview followed by a standardized physical examination in a mobile examination center (MEC). A stratified multistage sampling design with a weighting scheme based on the selection of counties, blocks, households, and persons within households is used by NHANES to represent the civilian, non-institutionalized population in the United States and accurately estimate the prevalence of diseases. Information about data analysis can be found on the web link: https://wwwn.cdc.gov/nchs/nhanes/tutorials/module2.aspx.

A total of 1,404 adults aged ≥60 years old who received the examinations of TG, HDL-C, and hand grip strength were initially included. Those who had surgeries on their hands or wrists were excluded, and finally, 1,306 of them were eligible. Since NHANES was approved by the Institutional Review Board (IRB) of the NCHS of the Centers for Disease Control and Prevention (CDC) of the United States, the data were publicly available; no ethical approval from the IRB of our institution was required.

### Calculation of serum TG/HDL-C ratio

The information on serum TG (mg/dL) and HDL-C (mg/dL) concentrations was extracted from the database. More information on laboratory examinations was provided on the website of the CDC (17). The TG/HDL-C ratios were calculated by dividing TG by HDL-C. In our analysis, we divided the TG/HDL-C ratio into three levels according to the tertiles, of which the 33% cutoff value was 1.47 and the 66% cutoff value was 2.69.

### Measurement of the relative grip strength

The relative grip strength was defined as the sum of the largest reading from each hand to the body mass index (BMI) ratio. In NHANES, hand grip strength (kg) was evaluated using the Hand Dynamometer (Takei Digital gripper force gage model T.K.K.5401), to obtain the maximum force exerted by hands. Further details on the grip strength test procedures are available elsewhere (18).

### Collection of variables

BMI was calculated as a ratio of weight in kilograms over height in meters squared (kg/m^2^), and participants were grouped into four categories: underweight (BMI < 18.50 kg/m^2^), normal (BMI = 18.50–24.99 kg/m^2^), overweight (BMI = 25.00–29.99 kg/m^2^), and obese (BMI ≥30.00 kg/m^2^) using the criteria of World Health Organization (WHO) for grouping weight status (19). We collected variables including age, gender, race, education level, marital status, income, smoking, drinking, physical activity, DM, hypertension, dyslipidemia, CVD, anemia, depression, osteoporosis, arthritis, chronic kidney disease (CKD), height (cm), weight (kg), serum albumin (g/dL), creatinine (mg/dL), white blood cell (WBC) (K/uL), protein (g) and its supplement, vitamin D (mcg) and its supplement, and polyunsaturated fatty acid (PUFA) (gm) and its supplement from the NHANES.

DM was defined as fasting blood glucose ≥7.0 mmol/L or glycosylated hemoglobin (HbAlc) ≥6.5% or self-reported DM or receiving hypoglycemic therapy (20). CVD was diagnosed according to the positive answer to the multiple-choice question (MCQ): ‘Have you ever been told you had (congestive) heart failure, coronary heart disease, angina/angina pectoris, heart attack, and stroke’ or if an individual is using cardiovascular drugs for any one or more diseases. Hypertension was defined as a mean blood pressure exceeding 140/90 mmHg for systolic pressure and diastolic pressure, respectively. Hyperlipidemia was classified as total cholesterol ≥200 mg/dL, triglycerides ≥150 mg/dL, HDL ≥ 40 mg/dL in men and ≥ 50 mg/dL in women, or low-density lipoprotein ≥130 mg/dL. Alternately, persons who reported using cholesterol-lowering drugs were also classified as having hyperlipidemia (21). Hemoglobin levels were categorized into non-anemia (≥12 g/dL) and anemia (<12 g/dL). Depression was assessed using the Patient Health Questionnaire (PHQ-9) of the NHANES, and the PHQ score (range 0–27) cutoff point ≥ 10 showed that the sensitivity of diagnosing major depression is 88%, and the specificity is 88% (22). Osteoporosis was defined according to the World Health Organization (WHO) criteria (23). Arthritis status was determined by responses to the Medical Condition questionnaire based on which a doctor or health professional informed the individual that they had arthritis. Serum creatinine was used to calculate eGFR with the Chronic Kidney Disease Epidemiology Collaboration (CKD-EPI) equation (24). More information on these covariates can be found on the NHANES website [https://www.cdc.gov/nchs/nhanes/ (accessed on 20 April 2022)].

### Statistical analysis

Continuous data were described by mean ± standard error (mean ± SE), and the F test was used for comparison in groups. Categorical data were expressed as frequency with constituent ratio [N (%)], and the chi-square test was used for comparison. Because TG is measured in a fasting subsample of persons, special sample weights are required to analyze these data properly: the 2-year cycle of MEC exam weight (wtmec2yr). More details of the NHANES weights can be found online: https://wwwn.cdc.gov/Nchs/Nhanes/2013-2014/TRIGLY_H.htm.

Weighted univariate linear regression and stepwise regression analyses were used to screen the covariates. Weighted univariate and multivariate linear regression analyses were used to explore the association between the TG/HDL-C ratio and the relative grip strength. Model 1 was the crude model. Model 2 adjusted for demographic variables including age, gender, race, and marital status. Model 3 additionally adjusted for covariates including physical activity, DM, CVD, arthritis, and albumin. We also explored this relationship in subgroups of gender, DM, CVD, and arthritis. The evaluation indexes were β and 95% confidence intervals (CIs). Two-sided *p* < 0.05 was considered significant. Statistical analysis was performed using SAS 9.4 (SAS Institute, Cary, NC, United States). Missing variables (including education level, serum creatinine, albumin, and WBC) were interpolated using the random forest method, and sensitivity analysis was performed on the characteristics of participants before and after the imputation (Supplementary Table S1).

## Results

### Characteristics of participants

**Figure 1:**
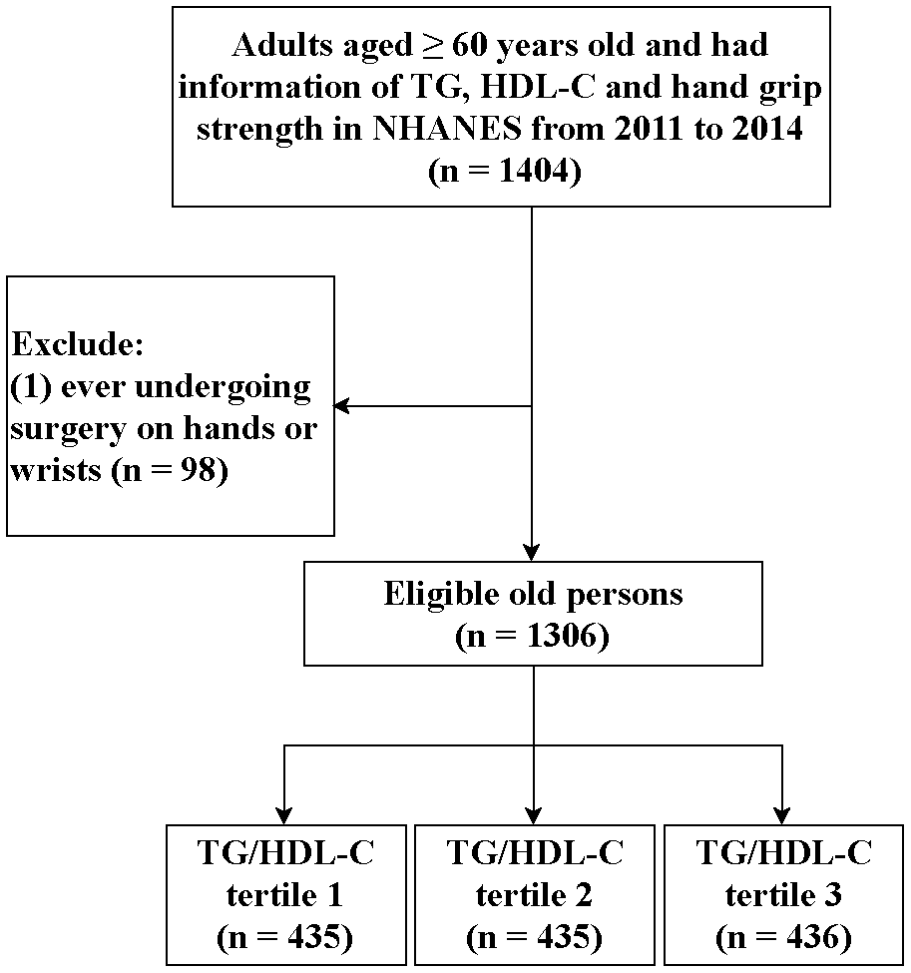
Flow chart of the screening of participants.

The flowchart of the screening of participants is presented in Figure 1. A total of 1,404 individuals aged ≥60 years old with information on TG, HDL-C, and hand grip strength in the NHANES were initially included. Then, we excluded those who underwent surgery on their hands or wrists (*n* = 98), and finally, 1,306 of them were eligible. Participants were divided into three groups according to the TG/HDL-C levels: tertile 1 (*n* = 435), tertile 2 (*n* = 435), and tertile 3 (*n* = 436).

The characteristics of participants are presented in Table 1. The average age of the study population was 69.07 years old, and 648 (54.22%) of them were women. Most of them do not have DM [816 (69.62%)], CVD [721 (57.13%)], or CKD [1,037 (81.29%)]. In total, 1,068 (79.31%) individuals have hypertension, and 1,117 (85.21%) have dyslipidemia. The mean TG/HDL-C ratio and relative grip strength were 2.60 and 2.21, respectively. Additionally, race, education level, income, drinking, height, weight, BMI, WBC, vitamin D, TG, and HDL-C were all significantly different among the three tertile groups (all *p* < 0.05).

**Table 1:** The characteristics of participants.

Variables	Total (*n* = 1,306)	TG/HDL-C ratio			Statistics	*p*-value
		Tertile 1 (*n* = 435)	Tertile 2 (*n* = 435)	Tertile 3 (*n* = 436)		
Age, years, mean (S.E)	69.07 (0.26)	68.70 (0.48)	69.67 (0.31)	68.90 (0.38)	*F* = 3.44	0.045
Gender, *n* (%)					χ^2^ = 4.000	0.135
Men	658 (45.78)	198 (41.37)	215 (45.27)	245 (50.78)		
Women	648 (54.22)	237 (58.63)	220 (54.73)	191 (49.22)		
Race, *n* (%)					χ^2^ = 41.869	<0.001
Mexican American	117 (3.67)	19 (1.58)	52 (5.24)	46 (4.37)		
Non-Hispanic Black	282 (8.94)	130 (12.02)	89 (9.28)	63 (5.47)		
Non-Hispanic White	637 (78.50)	215 (80.08)	202 (76.51)	220 (78.70)		
Other Hispanic	133 (3.56)	26 (1.91)	49 (4.20)	58 (4.66)		
Other Race - Including Multi-Racial	137 (5.33)	45 (4.41)	43 (4.77)	49 (6.80)		
Education level, *n* (%)					χ^2^ = 28.163	<0.001
Less than 9th grade	179 (7.39)	40 (4.89)	69 (8.88)	70 (8.59)		
9–11th grade (Includes 12th grade with no diploma)	199 (11.15)	62 (8.87)	69 (13.99)	68 (10.90)		
High school graduate/GED or equivalent	312 (22.80)	94 (19.32)	109 (24.17)	109 (25.11)		
College graduate or above	277 (28.10)	128 (40.10)	79 (22.36)	70 (21.05)		
Some college or AA degree	339 (30.55)	111 (26.82)	109 (30.60)	119 (34.36)		
Marital status, *n* (%)					χ^2^ = 0.138	0.933
Married	756 (65.39)	239 (64.63)	264 (66.31)	253 (65.33)		
Unmarried	550 (34.61)	196 (35.37)	171 (33.69)	183 (34.67)		
Income, *n* (%)					χ^2^ = 13.563	0.001
<$20,000	347 (16.27)	90 (10.17)	121 (20.35)	136 (18.78)		
≥$20,000	959 (83.73)	345 (89.83)	314 (79.65)	300 (81.22)		
Smoking, *n* (%)					χ^2^ = 5.121	0.077
No	880 (72.42)	299 (76.56)	304 (74.02)	277 (66.68)		
Yes	426 (27.58)	136 (23.44)	131 (25.98)	159 (33.32)		
Drinking, *n* (%)					χ^2^ = 6.314	0.043
No	429 (28.14)	129 (25.34)	157 (34.70)	143 (24.97)		
Yes	877 (71.86)	306 (74.66)	278 (65.30)	293 (75.03)		
Physical activity, *n* (%)					χ^2^ = 5.424	0.066
High level	424 (37.31)	156 (43.42)	135 (34.47)	133 (33.64)		
Low level	882 (62.69)	279 (56.58)	300 (65.53)	303 (66.36)		
DM, *n* (%)					χ^2^ = 76.866	<0.001
No	816 (69.62)	326 (84.56)	276 (67.41)	214 (56.27)		
Yes	490 (30.38)	109 (15.44)	159 (32.59)	222 (43.73)		
Hypertension, *n* (%)					χ^2^ = 10.150	0.006
No	238 (20.69)	92 (26.28)	88 (22.68)	58 (13.12)		
Yes	1,068 (79.31)	343 (73.72)	347 (77.32)	378 (86.88)		
Dyslipidemia, *n* (%)					χ^2^ = 67.048	<0.001
No	189 (14.79)	119 (29.29)	64 (12.97)	6 (1.54)		
Yes	1,117 (85.21)	316 (70.71)	371 (87.03)	430 (98.46)		
CVD, *n* (%)					χ^2^ = 14.057	<0.001
No	721 (57.13)	261 (64.57)	249 (58.03)	211 (48.65)		
Yes	585 (42.87)	174 (35.43)	186 (41.97)	225 (51.35)		
Anemia, *n* (%)					χ^2^ = 0.527	0.769
No	1,127 (91.36)	368 (91.64)	376 (90.36)	383 (91.98)		
Yes	179 (8.64)	67 (8.36)	59 (9.64)	53 (8.02)		
Depression, *n* (%)					χ^2^ = 3.740	0.154
No	1,204 (93.10)	408 (93.26)	406 (95.11)	390 (91.09)		
Yes	102 (6.90)	27 (6.74)	29 (4.89)	46 (8.91)		
Osteoporosis, *n* (%)					χ^2^ = 1.300	0.522
No	1,218 (93.18)	401 (91.85)	406 (93.25)	411 (94.48)		
Yes	88 (6.82)	34 (8.15)	29 (6.75)	25 (5.52)		
Arthritis, *n* (%)					χ^2^ = 0.149	0.928
No	708 (52.95)	224 (52.19)	250 (54.04)	234 (52.72)		
Yes	598 (47.05)	211 (47.81)	185 (45.96)	202 (47.28)		
CKD, *n* (%)					χ^2^ = 13.312	0.001
No	1,037 (81.29)	373 (87.95)	340 (78.64)	324 (76.88)		
Yes	269 (18.71)	62 (12.05)	95 (21.36)	112 (23.12)		
Height, cm, mean (S.E)	166.82 (0.44)	165.63 (0.47)	166.64 (0.68)	168.20 (0.81)	*F* = 3.53	0.041
Weight, kg, mean (S.E)	81.19 (1.21)	73.19 (1.23)	80.70 (1.32)	89.89 (1.90)	*F* = 34.42	<0.001
BMI, kg/m^2^, mean (S.E)	29.07 (0.39)	26.61 (0.38)	29.03 (0.55)	31.65 (0.54)	*F* = 42.18	<0.001
BMI, *n* (%)					χ^2^ = 80.355	<0.001
Obesity	474 (36.98)	106 (19.81)	149 (37.16)	219 (54.51)		
Overweight	467 (35.98)	146 (36.29)	169 (38.02)	152 (33.79)		
Underweight and normal	365 (27.03)	183 (43.90)	117 (24.82)	65 (11.70)		
Albumin, g/dL, mean (S.E)	4.21 (0.01)	4.21 (0.02)	4.21 (0.02)	4.20 (0.02)	*F* = 0.25	0.783
Creatinine, mg/dL, mean (S.E)	0.97 (0.02)	0.95 (0.05)	0.96 (0.02)	1.00 (0.02)	*F* = 0.87	0.429
WBC, K/uL, mean (S.E)	6.61 (0.11)	6.10 (0.16)	6.47 (0.12)	7.27 (0.15)	*F* = 19.04	<0.001
Protein, g, mean (S.E)	72.21 (1.44)	74.29 (2.23)	71.14 (2.48)	71.06 (2.47)	*F* = 1.01	0.375
Protein supplement, g, mean (S.E)	0.07 (0.04)	0.13 (0.11)	0.04 (0.01)	0.03 (0.01)	*F* = 1.07	0.354
PUFA, gm, mean (S.E)	16.69 (0.53)	17.68 (0.70)	16.15 (0.58)	16.15 (1.11)	*F* = 2.84	0.073
PUFA supplement, gm, mean (S.E)	0.10 (0.02)	0.12 (0.03)	0.12 (0.03)	0.07 (0.01)	*F* = 1.56	0.225
Vitamin D, mcg, mean (S.E)	4.87 (0.13)	5.48 (0.31)	4.68 (0.28)	4.42 (0.26)	*F* = 3.48	0.043
Vitamin D supplement, mcg, mean (S.E)	20.48 (1.19)	22.07 (2.25)	21.60 (2.32)	17.82 (2.26)	*F* = 1.19	0.316
TG, mg/dL, mean (S.E)	124.66 (4.45)	66.76 (1.19)	109.74 (1.66)	197.99 (5.28)	*F* = 484.71	<0.001
HDL-C, mg/dL, mean (S.E)	56.66 (1.14)	71.58 (1.05)	54.34 (0.60)	43.45 (0.68)	*F* = 515.21	<0.001
TG/HDL-C ratio, mean (S.E)	2.60 (0.15)	0.97 (0.02)	2.03 (0.03)	4.81 (0.18)	*F* = 699.66	<0.001
Grip strength, kg, mean (S.E)	61.89 (0.80)	60.37 (1.29)	60.87 (1.28)	64.40 (1.62)	*F* = 1.70	0.199
Relative grip strength, mean (S.E)	2.21 (0.03)	2.33 (0.04)	2.18 (0.06)	2.10 (0.06)	*F* = 5.96	0.006

F, F test; χ^2^, chi-square test.

TG, triglyceride; HDL-C, high-density lipoprotein cholesterol; SE, standard error; DM, diabetes mellitus; CVDs, cardiovascular disease; CKDs, chronic kidney diseases; BMI, body mass index; WBC, white blood cell; PUFA, polyunsaturated fatty acid.

Tertile 1: TG/HDL-C ratio < 1.47.

Tertile 2: 1.47 ≤ TG/HDL-C ratio < 2.69.

Tertile 3: TG/HDL-C ratio ≥ 2.69.

### Association between TG/HDL-C ratio and the relative grip strength

Table 2 shows the covariates that are associated with the relative grip strength. We found that age, gender, race, education level, marital status, income, drinking, physical activity, DM, hypertension, dyslipidemia, CVD, osteoporosis, arthritis, CKD, albumin, WBC, protein, and PUFA were associated with relative grip strength (all *p* < 0.05). Then, we included these variables in a stepwise regression analysis, and the results showed that age, gender, race, marital status, physical activity, DM, CVD, arthritis, CKD, and albumin were the final covariates.

**Table 2:** Screening of covariates for the relative grip strength.

Variables	Univariate linear regression		Stepwise regression	
	β (95% CI)	*p*	β (95% CI)	*p*
Age	−0.03 (−0.04, −0.02)	<0.001	−0.02 (−0.02, −0.01)	<0.001
**Gender**				
Men	Ref		Ref	
Women	−1.03 (−1.12, −0.95)	<0.001	−0.98 (−1.07, −0.89)	<0.001
**Race**				
Mexican American	Ref		Ref	
Non-Hispanic Black	0.37 (0.17, 0.56)	<0.001	0.37 (0.27, 0.47)	<0.001
Non-Hispanic White	0.33 (0.12, 0.55)	0.003	0.21 (0.12, 0.30)	<0.001
Other Hispanic	0.06 (−0.18, 0.31)	0.599	0.04 (−0.05, 0.12)	0.364
Other Race - Including Multi-Racial	0.29 (0.06, 0.51)	0.016	0.21 (0.10, 0.32)	0.001
**Education level**				
9–11th grade (Includes 12th grade with no diploma)	Ref			
College graduate or above	0.43 (0.26, 0.60)	<0.001		
High school graduate/GED or equivalent	0.01 (−0.14, 0.16)	0.938		
Less than 9th grade	−0.08 (−0.26, 0.10)	0.376		
Some college or AA degree	0.07 (−0.08, 0.21)	0.354		
**Marital status**				
Married	Ref		Ref	
Unmarried	−0.36 (−0.50, −0.21)	<0.001	−0.1 (−0.19, −0.01)	0.025
**Income**				
<$20,000	Ref			
≥$20,000	0.38 (0.26, 0.51)	<0.001		
**Smoking**				
No	Ref			
Yes	0 (−0.11, 0.12)	0.942		
**Drinking**				
No	Ref			
Yes	0.44 (0.30, 0.57)	<0.001		
**Physical activity**				
High level	Ref		Ref	
Low level	−0.5 (−0.64, −0.37)	<0.001	−0.24 (−0.35, −0.13)	<0.001
**DM**				
No	Ref		Ref	
Yes	−0.39 (−0.49, −0.29)	<0.001	−0.21 (−0.27, −0.15)	<0.001
**Hypertension**				
No	Ref			
Yes	−0.22 (−0.41, −0.03)	0.028		
**Dyslipidemia**				
No	Ref			
Yes	−0.39 (−0.57, −0.20)	<0.001		
**CVD**				
No	Ref		Ref	
Yes	−0.18 (−0.32, −0.03)	0.020	−0.13 (−0.22, −0.04)	0.007
**Osteoporosis**				
No	Ref			
Yes	−0.29 (−0.50, −0.08)	0.008		
**Arthritis**				
No	Ref		Ref	
Yes	−0.39 (−0.51, −0.26)	<0.001	−0.17 (−0.28, −0.06)	0.004
**CKD**				
No	Ref			
Yes	−0.3 (−0.42, −0.18)	<0.001		
Albumin	0.65 (0.41, 0.88)	<0.001	0.41 (0.26, 0.57)	<0.001
Creatinine	0.09 (−0.09, 0.28)	0.311		
WBC	−0.04 (−0.07, −0.01)	0.007		
Protein	0.01 (0.01, 0.01)	<0.001		
Vitamin D	0 (−0.00, 0.00)	0.083		
PUFA	0.02 (0.01, 0.03)	<0.001		

CI, confidence interval; Ref, reference; DM, diabetes mellitus; CVD, cardiovascular disease; CKD, chronic kidney diseases; WBC, white blood cell; PUFA, polyunsaturated fatty acid.

After adjusting for the covariates above, we found that higher TG/HDL-C level was negatively linked to the relative grip strength [tertile 2: β = −0.1, 95% CI: (−0.19, −0.01); tertile 3: β = −0.19, 95% CI: (−0.26, −0.12)], indicating that the relative grip strength may decrease significantly along with the increase in the TG/HDL-C ratio (Table 3).

**Table 3:** Association between TG/HDL-C and the relative grip strength.

TG/HDL-C ratio	Model 1		Model 2		Model 3	
	β (95% CI)	*p*	β (95% CI)	*p*	β (95% CI)	*p*
Tertile 1	Ref		Ref		Ref	
Tertile 2	−0.16 (−0.30, −0.01)	0.036	−0.16 (−0.26, −0.05)	0.005	−0.1 (−0.19, −0.01)	0.028
Tertile 3	−0.23 (−0.37, −0.08)	0.003	−0.3 (−0.39, −0.21)	<0.001	−0.19 (−0.26, −0.12)	<0.001

TG, triglyceride; HDL-C, high-density lipoprotein cholesterol; CI, confidence interval; Ref, reference.

Model 1: crude model.

Model 2: adjusted for age, gender, race, and marital status.

Model 3: adjusted for age, gender, race, marital status, physical activity, DM, CVD, arthritis, CKD, and albumin.

Tertile 1: TG/HDL-C ratio < 1.47.

Tertile 2: 1.47 ≤ TG/HDL-C ratio < 2.69.

Tertile 3: TG/HDL-C ratio ≥ 2.69.

### Association between TG/HDL-C ratio and the relative grip strength in gender, DM, CVD, and arthritis subgroups

**Figure 2:**
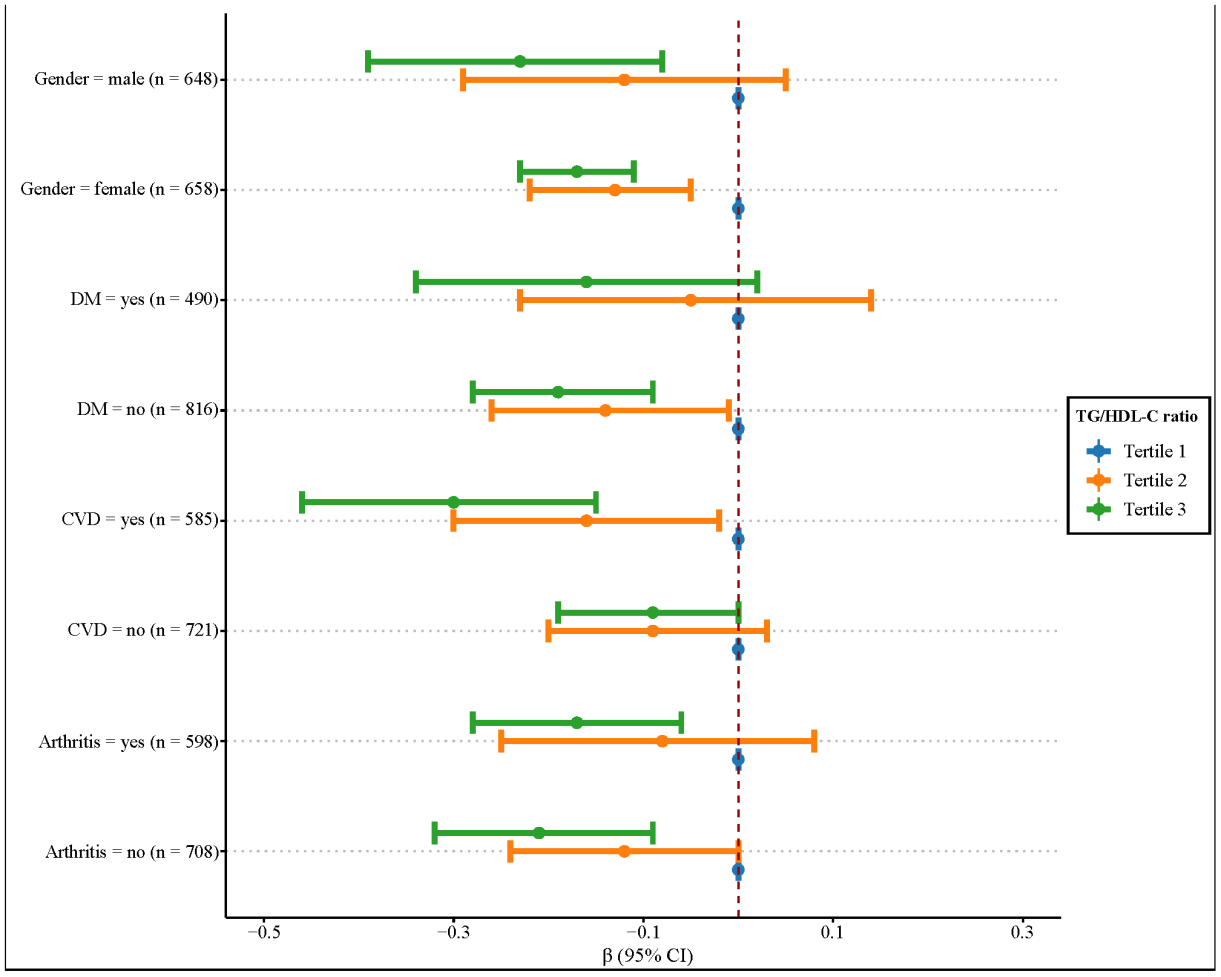
Association between the TG/HDL-C ratio and the relative grip strength in gender, DM, CVD, and arthritis subgroups.

We further explored this relationship in subgroups of gender, DM, CVD, and arthritis (Figure 2). The TG/HDL-C ratio was also negatively associated with the relative grip strength in participants who were women, with CVD, and without DM (all *p* < 0.05). In men, both arthritis and non-arthritis populations, this negative relationship only existed when the TG/HDL-C ratio was ≥2.69 (all *p* < 0.05).

## Discussion

In this study, we explored the association between the TG/HDL-C ratio and the relative grip strength in older adults. The results showed that after adjusting for the covariates including age, gender, race, marital status, physical activity, DM, CVD, arthritis, CKD, and albumin, the TG/HDL-C ratio was negatively associated with the relative grip strength. This relationship was also found in those who were women, not having DM, and having CVD.

The TG/HDL-C ratio can reflect the comprehensive level of lipid metabolism in the body and has been indicated as a risk factor in some diseases (25–27). Liu et al. (11) found that an increased TG/HDL-C ratio indicated a greater risk of new-onset T2DM and may be a simple but effective indicator in predicting T2DM in older adults. Wu et al. (28) showed that the TG/HDL-C ratio was significantly associated with arterial stiffness progression in hypertensive population. Kim et al. (29) considered that higher TG/HDL-C ratios were positively associated with CKD development in men, while middle levels of TG/HDL ratios reduced the CKD incidence in women. However, to the best of our knowledge, no previous study has explored the relationship between the TG/HDL-C ratio and grip strength. A MONET study on overweight and obese postmenopausal women showed that the TG/HDL-C ratio was significantly correlated with muscle strength (30). Wang et al. (14) found a negative association between sarcopenia and TG/HDL-C ratio in community-dwelling adults in China. In addition, Lin et al. (31) studied older patients with DM in China and found that there was a negative association between TG/HDL-C ratio and sarcopenia, indicating that a higher TG/HDL-C ratio was associated with better muscle status. Differently, in the current study, we found that the TG/HDL-C ratio was negatively associated with the relative grip strength in adults aged ≥60 years old, which indicated that the TG/HDL-C ratio may be suggested to be controlled at an appropriate level in clinical practice and may benefit to prevent and control sarcopenia in older adults. However, the causal association between the TG/HDL-C ratio and grip strength and the role of the TG/HDL-C ratio in sarcopenia needs further exploration.

The potential mechanism to explain the role of the TG/HDL-C ratio in grip strength and sarcopenia in older adults has been unclear. Based on the data from the 2019 Korea National Health and Nutrition Examination Survey, a study showed that TG was significantly lower while HDL-C was significantly higher in the participants with high muscle strength than in those with low muscle strength (32). Under healthy conditions, intramuscular TG can be utilized as a readily available fuel source for muscle contraction during physical exercise; however, it can also be associated with pathological conditions, such as IR and sarcopenia (33). In skeletal muscle, lipids in the plasma membrane can induce intracellular signal transduction such as releasing Ca^2+^ from the endoplasmic reticulum (ER), which has an important function in muscle contraction (34). A study conducted by Park et al. (35) showed that when the level of physical activity increased, the prevalence of sarcopenia decreased, and above moderate-active physical activity levels were associated with higher HDL-C than low-active physical activity. In addition, patients with T2DM are prone to lose muscle mass more rapidly, thus becoming more susceptible to IR than the general population (36). Therapeutics for patients with T2DM or other dysmetabolic conditions may affect the body composition, for example, the muscle strength and the muscle quality index (37). According to these findings, we speculated that disturbance in lipid metabolism including an increase in TG level and decrease in HDL-C level may contribute to reduced muscle strength during aging, which played an important role in the development of sarcopenia. In addition, physical activities in older adults can help improve musculoskeletal health by increasing muscle strength and flexibility, which are factors related to sarcopenia. We concluded that older adults should control TG/HDL-C levels within the appropriate range and appropriately strengthen exercise to prevent the occurrence of sarcopenia under the guidance of physicians.

We further explored the association between TG/HDL-C ratio and relative grip strength in gender, DM, CVD, and arthritis subgroups. Subgroup analyses showed that increased TG/HDL-C ratio was associated with decreased relative grip strength in older adults who were women, without DM, or with CVD. Gender is a risk factor for sarcopenia (38). A previous study indicated that muscle strength correlates with total body bone mineral density, which was significant in women but not in men (39). Kim et al. (40) investigated the changes in metabolic indices of serum lipid level and serum glucose level according to grip strength in postmenopausal women and found that with a high relative grip strength, the TG level decreased while the HDL-C level increased. The difference in the age-related changes in factors such as physical activity, nutritional status, sex hormones, and growth hormones which affect muscle quality/quantity between women and men may contribute to the gender difference in muscle (41). Insulin plays an important role in the anabolic action of skeletal muscle, and it can be progressively lost in T2DM, which may induce decreased protein synthesis and increased protein degradation, leading to a reduction in muscle strength (42). Increased TG/HDL-C ratio indicating a greater risk of new-onset T2DM is well known so DM patients may follow doctor’s advice or spontaneously control their TG and HDL-C levels through medication or other means (11). This may explain why this relationship was more significant among the non-DM population. CVD is a chronic comorbidity that often exacerbates sarcopenia (43). Studies have reported an increased prevalence of CVD risk factors, such as DM, hypertension, dyslipidemia, and metabolic syndrome, which are related to lowered skeletal muscle mass (44, 45). In addition, the TG/HDL-C ratio was associated with arterial stiffness progression in patients with hypertension (28). The underlying mechanisms of the relationship between sarcopenia and CVD are multifactorial, involving pathophysiological changes such as muscle mitochondria dysfunction, oxidative stress, multiple metabolic disorders, malnutrition, and insufficient physical activity (46, 47). Our results may be a supplement to previous reports, and we found that in older adults who had CVD, the TG/HDL-C ratio was negatively associated with the relative grip strength, indicating that it was meaningful to focus on this ratio in patients with CVD to prevent sarcopenia. We concluded that serum TG/HDL-C ratio level should be taken into consideration for the evaluation and dynamic monitoring for grip strength and sarcopenia in older adults, especially those who have different clinical chronic diseases.

### Strengths and limitations

Data of the study population were extracted from the NHANES database, which used a multistage sampling design with a weighting scheme, and thus, the sample size was large and representative. To the best of our knowledge, it is the first time to explore the association between the TG/HDL-C ratio and relative grip strength in individuals aged ≥60 years old. However, there are still some limitations to this study. This study is a retrospective study so no causal association could be established. In addition, information regarding the muscle function-related index was not available in the NHANES, and further study exploring the TG/HDL-C ratio and muscle function in older adults is still needed.

## Conclusion

With the increase in the TG/HDL-C ratio, the relative grip strength of older adults decreased significantly, indicating that the TG/HDL-C ratio should be closely monitored in the older adult population because it may associated with the prevention and control of sarcopenia.

## Data availability statement

Publicly available datasets were analyzed in this study. This data can be found here: the NHANES database, https://wwwn.cdc.gov/nchs/nhanes/.

## Ethics statement

The requirement of ethical approval was waived by The First Affiliated Hospital of Traditional Chinese Medicine of Chengdu Medical College for the studies involving humans because Since NHANES was approved by the Institutional Review Board of the NCHS of the US Centers for Disease Control and Prevention and the data were publicly available, no ethical approval of our IRB was required. The studies were conducted in accordance with the local legislation and institutional requirements. The ethics committee/institutional review board also waived the requirement of written informed consent for participation from the participants or the participants’ legal guardians/next of kin because Since NHANES was approved by the Institutional Review Board of the NCHS of the US Centers for Disease Control and Prevention and the data were publicly available, no ethical approval of our IRB was required.

## Author contributions

YH designed the study, wrote the manuscript, critically reviewed, edited, and approved the manuscript. JL and YL collected, analyzed, and interpreted the data. All authors contributed to the article and approved the submitted version.
