# Supplementary material for: Triglyceride to high-density lipoprotein cholesterol ratio was negatively associated with relative grip strength in older adults: a cross-sectional study of the NHANES database
Source: Front Public Health. 2023 Nov 2;11:1222636. doi: 10.3389/fpubh.2023.1222636 (PMC10656816; doi:10.3389/fpubh.2023.1222636)
Supplement: Supplementary file 1 [file Table_1.docx]

**Table S1. Sensitivity analysis before and after the random forest interpolation of missing data**

| Variables | Before interpolation (n=1306) | After interpolation (n=1306) | Statistics | *P* |
| --- | --- | --- | --- | --- |
| Education level, n (%) |  |  | χ^2^=7.711 | 0.103 |
| 9-11th grade (Includes 12th grade with no diploma) | 199 (11.15) | 199 (11.16) |  |  |
| College graduate or above | 277 (28.10) | 277 (28.12) |  |  |
| High school graduate/GED or equivalent | 312 (22.80) | 310 (22.76) |  |  |
| Less than 9th grade | 179 (7.39) | 179 (7.40) |  |  |
| Some college or AA degree | 339 (30.55) | 339 (30.57) |  |  |
| Income, n (%) |  |  | χ^2^=1.637 | 0.201 |
| < $20000 | 347 (16.27) | 341 (16.43) |  |  |
| ≥ $20000 | 959 (83.73) | 896 (83.57) |  |  |
| Drinking, n (%) |  |  | χ^2^=1.025 | 0.311 |
| No | 429 (28.14) | 419 (28.34) |  |  |
| Yes | 877 (71.86) | 835 (71.66) |  |  |
| Height, cm, Mean (S.E) | 166.82 (0.44) | 166.80 (0.44) | t=0.78 | 0.443 |
| Weight, kg, Mean (S.E) | 81.19 (1.21) | 81.20 (1.22) | t=-1.35 | 0.186 |
| BMI, kg/m^2^, Mean (S.E) | 29.07 (0.39) | 29.06 (0.39) | t=0.97 | 0.338 |
| Albumin, g/dL, Mean (S.E) | 4.21 (0.01) | 4.21 (0.01) | t=-1.53 | 0.135 |
| Creatinine, mg/dL, Mean (S.E) | 0.97 (0.02) | 0.97 (0.02) | t=-1.45 | 0.156 |
| WBC, K/uL, Mean (S.E) | 6.61 (0.11) | 6.61 (0.11) | t=1.01 | 0.320 |

t: t test, χ^2^: chi-square test

SE: standard error, BMI: body mass index, WBC: white blood cell
